# Gut microbiota profile in school-aged children with vitamin A depletion and vitamin E deficiency: A cross-sectional study

**DOI:** 10.1080/29933935.2026.2747503

**Published:** 2026-10-16

**Authors:** Blanca Estela López-Contreras, Paola León-Mimila, Hugo Villamil-Ramírez, Sofía Morán-Ramos, Daniel Cerqueda-García, Blanca E. Del-Río-Navarro, Carlos A. Aguilar-Salinas, Teresa Villarreal-Molina, Mariela Camacho-Barrón, Olga P. García, Samuel Canizales-Quinteros

**Affiliations:** a Unidad Periférica de Investigación en Genómica de Poblaciones Aplicada a la Salud, Departamento de Biología, Facultad de Química, Universidad Nacional Autónoma de México (UNAM)/Instituto Nacional de Medicina Genómica (INMEGEN), Mexico City, México; b Departamento de Alimentos y Biotecnología, Facultad de Química, UNAM, Mexico City, México; c Red de Manejo Biorracional de Plagas y Vectores, Instituto de Ecología, Clúster Científico y Tecnológico BioMimic, A.C.-INECOL, Veracruz, México; d Laboratorio de Investigación de Alergia e Inmunología, Hospital Infantil de México Federico Gómez, Mexico City, México; e Unidad de Investigación de Enfermedades Metabólicas/Departamento de Endocrinología y Metabolismo de Lípidos, Instituto Nacional de Ciencias Médicas y Nutrición Salvador Zubiran (INCMNSZ), Mexico City, México; f Laboratorio de Genómica Cardiovascular, Instituto Nacional de Medicina Genómica (INMEGEN), Mexico City, México; g Facultad de Ciencias Naturales, Universidad Autónoma de Querétaro (UAQ), Querétaro, México

**Keywords:** Vitamin A depletion, vitamin E deficiency, children, gut microbiota, diet

## Abstract

Vitamin A (VitA) and vitamin E (VitE) deficiencies can result in significant health consequences in children. The role of the intestinal microbiota in these vitamin deficiencies has been scarcely explored. This cross-sectional study aimed to analyze the relationship of VitA depletion and VitE deficiency with metabolic traits, dietary intake, and the gut microbiota composition in 179 school-age children. Serum VitA depletion and VitE deficiency were present in 8.4% and 20.7% of children, respectively. No significant relationship between tocopherol or retinol intake and VitA depletion or VitE deficiency was found. Linear discriminant analysis effect size (LEfSe) and analysis of compositions of microbiomes with bias correction 2 (ANCOM-BC2) analysis revealed no differences in the gut microbiota composition according to VitE status. However, we observed significant enrichment of *Veillonella*, *Haemophilus,* and *Streptococcus* in children with VitA depletion as compared to those with normal VitA levels. The enrichment of these oropharyngeal genera in VitA depletion resembles the gut microbiome composition previously described in childhood stunting. Future functional studies are required to elucidate whether these bacteria play a role in vitamin A metabolism.

## Introduction

VitA and VitE are essential liposoluble vitamins that regulate metabolic health, as well as antioxidant and immune responses.^1^ Deficiencies in VitA and VitE may occur in individuals with malnutrition and metabolic dysfunctions, such as obesity, diabetes, and dyslipidemia.^2-4^ Mechanistic evidence from cellular and rodent models has suggested that this depletion drives systemic low-grade inflammation and insulin resistance through dual pathways. First, at the adipose tissue level, VitA depletion stimulates adipogenesis through PPARg activation and alters adipokine and uncoupling protein expression, thereby lowering energy expenditure. Furthermore, this deficiency shifts the immune profile by exacerbating a Th1-type response, which promotes macrophage infiltration.^4-6^ Second, depletion damages epithelial barrier integrity. Specifically, VitE depletion impairs the expression of occludins that play a key role in the maintenance of tight junctions. Meanwhile, VitA deficiency disturbs the activity of enterocyte enzymes of the intestinal barrier, triggering bacterial translocation and endotoxemia.^7-9^

In Mexico, despite high rates of overweight and obesity, one in four school-aged children suffers from micronutrient deficiencies.^10-12^ In this regard, studies have shown that dietary intake correlates weakly with circulating vitamin levels;^3^ ^,^ ^13^ ^,^ ^14^ therefore, identifying other biological factors affecting their bioavailability is crucial. Recent studies in model organisms and humans have demonstrated that the gut microbiota plays a relevant role in the bioavailability, biosynthesis and biotransformation of molecules such as vitamins.^15^ ^,^ ^16^ Significant associations between the serum levels of certain vitamins and gut microbiome species have been described.^17^ Other studies have reported the role of the gut microbiota in micronutrient absorption and metabolism. Specifically, gut microbiota-derived secondary bile acids are required for the absorption of liposoluble vitamins A and E. Dysbiosis could thus lead to impaired absorption and consequently vitamin deficiencies.^18^ ^,^ ^19^ Moreover, a Mendelian randomization study suggested a causal relationship between certain taxonomic groups and vitamin deficiencies.^20^

Therefore, we hypothesized that VitA depletion and VitE deficiency in Mexican school-aged children are associated with metabolic dysfunction, a proinflammatory state, and distinct gut microbiota profiles. Consequently, this study aimed to evaluate the risk of VitA and VitE deficiencies and their relationships with metabolic parameters and the gut microbiota composition in Mexican school-aged children.

## Methods

### Study cohort

This cross-sectional observational study is nested in the Obesity Research Study of Mexican Children, which is a population-based study in Mexico City.^21^ We included children recruited during July of 2015, reducing confounders environmental factors, such as dietary seasonality and infectious disease rates.^22^ A subset of 179 children (88 boys and 91 girls) aged 6–12 years was selected based on the following data availability: (1) validated dietary records, (2) VitA and VitE quantification, and (3) valid 16S rRNA gene sequencing data after quality control. Subjects with missing data were excluded.

Height, weight, waist, and hip circumferences were measured following standard protocols and calibrated instruments. BMI was calculated as body weight in kilograms divided by the square of height in meters (kg/m^2^). The BMI percentile was calculated as recommended by the Centers for Disease Control and Prevention using age and sex-specific BMI reference data.^23^

This study was conducted according to the Declaration of Helsinki and was approved by the Ethics Committee of the Instituto Nacional de Medicina Genómica (CEI 2015/01) and Hospital Infantil de Mexico (HIM 2017-090). The parents or guardians of each child signed the informed consent form, and the children assented to participate in the protocol.

### Biochemical measurements

Venous blood samples were drawn after 8–12 h of overnight fasting. Biochemical determinations, including glucose, total cholesterol, HDL cholesterol, and triglycerides (TG), were determined in the serum using standardized enzymatic methods (UNICEL DxC600, Beckman Coulter). Serum insulin concentrations were determined by radioimmunoassay (Beckman Coulter). Insulin resistance was estimated with the Homeostatic Model Assessment for Insulin Resistance (HOMA-IR).^24^ Serum high-sensitivity C-reactive protein (hsCRP) concentrations were determined by immunonephelometry (Cardiophase hsCRP, SIEMENS Healthcare Diagnostics Products GmbH, Marburg, Germany) on a BN ProSpec nephelometer according to the manufacturer's procedures.

### Plasma vitamin measurements

Plasma concentrations of VitE (alpha-tocopherol) and VitA (retinol) were measured at the Human Nutrition Laboratory at the Universidad Autónoma de Querétaro by reverse phase high-pressure liquid chromatography (HPLC) (Mod 2996, Waters Associates, Milford, MA, USA), at a wavelength of 300 nm with a C18 column (Waters, New Braunfels, TX, USA), using certified retinol and alpha-tocopherol standards, and a mobile phase of 100% methanol (J.T. Baker, Deventer, Netherlands) as previously described.^11^

Normal VitA status was defined as plasma retinol concentrations ≥30 μg/dL, risk of depletion <30 μg/dL, depletion <20 μg/dL, and deficiency <10 μg/dL according to the International VitA Consultative Group (IVACG) and NHANES-USA.^25^ Because only one child showed vitamin E deficiency based on a vitamin E:lipids ratio cutoff of 0.8,^26^ we defined vitamin E deficiency as a plasma *α*-tocopherol concentration <5 μg/mL.^26^ ^,^ ^27^

### Dietary intake assessment

The habitual diet was evaluated using a Food Frequency Questionnaire (FFQ) previously validated in a Mexican population.^28^ The FFQ was answered by parents of the participants to estimate their habitual dietary intake over the previous year. The FFQ included 116 food items and 10 frequency options: 6 or more per day, 4–5 per day, 2–3 per day, 1 per day, 5–6 per week, 2–4 per week, 1 per week, 2–3 per month, 1 per month or less, and never. Estimations of daily average energy, macro, and micronutrients were made through the Evaluation System of Nutritional Habits and Nutrient Intake software, based on the food composition tables compiled by the Instituto Nacional de Salud Pública as previously described.^29^ VitA activity in food was expressed as retinol activity equivalents (RAE), considering both preformed VitA (retinol) and provitamin A carotenoids.^30^

The US Dietary Reference Intakes were used to define the recommended dietary allowance (RDA) of vitamins A and E for children.^30^ ^,^ ^31^ The RDA for VitA is 400 μg/day of RAE for children ages 4–8 y and 600 μg/day for children ages 9–13 y. The RDA for VitE is 7 mg/day of *α*-tocopherol for children ages 4–8  y and 11 mg/day for children ages 9–13 y.

### Stool sampling, DNA extraction, and 16S rRNA sequencing

All the participants provided a fecal sample weighing between 3 and 8 g in a 50 ml sterile stool sample container without DNA preservation buffer. The samples remained refrigerated until delivery to the study staff and stored at −70 °C. Microbial DNA was extracted from a 200 mg aliquot using the QIAamp DNA Stool Mini Kit (Qiagen). The DNA concentration and purity were measured by spectrophotometry (Nanodrop 2000c). A PCR reaction containing 100 ng of DNA was performed using the 515F and 806R primers to amplify the V4 hypervariable region of the 16S rRNA. Purified amplicon concentrations were measured by fluorometry (Qubit 2.0, Invitrogen) to be pooled equimolarly and sequenced using the Illumina MiSeq 2 × 250 platform as previously described.^21^ ^,^ ^32^

### Gut microbiome characterization

The QIIME2 platform was used to analyze all 16S rRNA sequences.^33^ Denoising and the elimination of chimeras were performed using DADA2.^34^ The removal of chimeric sequences was carried out through the “consensus” method. The SILVA database v.132 was used as a reference for amplicon sequence variant (ASV) taxonomic assignment. Phylogeny was built using the “align-to-tree-mafft-fasttree” module.^35^ ^,^ ^36^ The samples were standardized for rarefaction at 12,000 high-quality read depth. The rarefaction depth of 12,000 reads per sample was selected after inspecting the ASV rarefaction curves (Supplementary Figure 1). At this sequencing depth, most samples showed a marked reduction in the slope of the accumulation curves, indicating that ASV richness was approaching a plateau and that additional sequencing depth would result in comparatively small increases in observed richness. This threshold, therefore, represented a balance between retaining sufficient sequencing depth for alpha- and beta-diversity analyses and avoiding the exclusion of samples due to lower read counts.

Data were exported to the R environment (R version 4.3.2) and analyzed using the phyloseq package (v.1.46.0). Alpha diversity was assessed by observed ASVs, Chao1, and Shannon indices, while beta diversity was calculated using weighted and unweighted UniFrac distances. Differences in alpha diversity indicators were adjusted by age, performing linear regression models (lm). The adonis2 function from the vegan package (v.2.7.1) and the pairwiseAdonis package were used to test differences in beta diversity measures, adjusting for age, using 9999 permutations (*p*-value < 0.05).

Differential abundance analysis was performed at the genus level using Linear Discriminant Analysis (LDA) effect size (LEfSe)^37^ and analysis of compositions of microbiomes with bias correction 2 (ANCOM-BC2) adjusted for age.^38^ LEfSe was performed using the MicrobiomeAnalyst 2.0 platform, following the default parameters for data filtering and the total sum scaling (TSS) method for data normalization. ANCOM-BC2 was performed using the R package ANCOMBC. Genus abundance was considered significantly differentiated according to the following criteria: (i) linear discriminant analysis (LDA) score ≥ 2 with *p*-value < 0.05 in LEfSe analysis; and (ii) Benjamini–Hochberg-adjusted *q-*value < 0.2 and passed sensitivity analysis for excess zeros in ANCOM-BC2, as previously reported.^39^

### Statistical methods

Anthropometric and metabolic characteristics of participants were compared using the median and interquartile range. Categorical variables were compared using the chi-square test. Differences in continuous variables among study groups were evaluated by non-parametric tests (Kruskal-Wallis or Mann-Whitney U). Linear regression models were adjusted for potential confounders: anthropometric variables were adjusted for sex and age, while biochemical parameters and vitamin levels were adjusted for sex, age, and BMI percentile. Partial Spearman correlations adjusted for potential confounders such as age, sex, and BMI percentile were used to test correlations of the abundance of bacteria differentiated by vitamin status with vitamin plasma levels and dietary intake. All the statistical analyses were performed using the SPSS Statistics package (IBM SPSS Statistics, version 24.0, Chicago, IL, USA). *P*-values less than 0.05 were considered statistically significant after false discovery rate (FDR) correction.

## Results

### Clinical, anthropometric, and biochemical characteristics in children with vitamin A depletion and vitamin E deficiency

In our study of Mexican children, 41.3% had normal, 50.3% had risk, and 8.4% had depletion of plasma VitA levels. Notably, none of the schoolchildren presented with VitA deficiency. Table 1 shows that children with risk and depletion were on average 1 y younger than those with normal VitA levels. However, only children with VitA depletion had significantly lower height percentile, body fat percentage, waist, waist to hip ratio (WHR), serum insulin, and TG levels as compared to the normal VitA groups (*p* < 0.05). All differences remained significant after adjusting for age, sex, and BMI percentile, except for insulin and triglyceride levels (*p*_adj_ = 0.633 and p_adj_ = 0.095, respectively). Of note, hsCRP levels were significantly higher in children with VitA depletion (*p* = 0.056) after adjusting for age, sex, and BMI percentile (*p*_adj_ = 0.006). Moreover, VitE deficiency was identified in 37 children (20.7%), and no differences in sex, age, and anthropometric parameters were observed between children with normal and deficient VitE levels. Only TG levels were significantly lower in children with VitE deficiency (*p*_adj_ = 0.001).

**Table 1.** Clinical and anthropometric characteristics stratified by vitamin A and vitamin E status.

		Vitamin A					Vitamin E		
	All (*n* = 179)	Normal (*n* = 74)	Risk (*n* = 90)	Depletion (*n* = 15)	*p-value* ^a^	*p-value* ^b^	Normal (*n* = 142)	Deficiency (*n* = 37)	*p-value* ^c^
Age (years)	9.7 (8.4–11.1)	10.2 (8.9–11.6)	9.3 (7.9–10.7)	9.3 (7.6–11.6)	0.005	0.117	9.3 (8.2–11.0)	10.1 (9.0–11.2)	0.209
Sex (girls, %)	91 (50.8)	39 (52.7)	45 (50.0)	7 (46.7)	0.730	0.670	73 (51.4)	18 (48.6)	0.765
Height percentile	48.9 (24.2–69.0)	52.9 (19.4–73.9)	49.8 (28.7–69.2)	32.6 (7.5–47.4)	0.843	0.030	49.3 (23.8–68.6)	48.4 (22.4–76.3)	0.693
BMI percentile	75.4 (36.2–93.3)	78.2 (41.1–93.1)	79.2 (30.8–94.1)	52.5 (14.3–79.3)	0.830	0.057	74.5 (30.2–93.1)	81.3 (43.6–95.2)	0.296
Body fat (%)	34.8 (26.3–42.4)	36.4 (27.4–42.8)	33.8 (26.8–42.2)	27.8 (21.8–33.8)	0.835	0.025	34.8 (26.0–41.8)	35.6 (28.7–44.2)	0.242
Waist (cm)	66.0 (57.0–76.0)	68.0 (60.8–76.6)	65.8 (56.4–76.0)	56.0 (51.5–69.0)	0.283	0.006	65.3 (56.9–75.0)	70.5 (57.0–77.5)	0.368
Hip (cm)	76.0 (69.0–86.0)	78.3 (71.0–88.5)	75.3 (68.0–86.4)	69.0 (63.5–80.0)	0.143	0.010	75.0 (68.8–86.0)	79.0 (69.0–88.5)	0.351
WHR	0.48 (0.44–0.54)	0.50 (0.44–0.55)	0.49 (0.44–0.56)	0.45 (0.42–0.47)	0.802	0.045	0.48 (0.44–0.54)	0.50 (0.45–0.55)	0.470
Glucose (mg/dL)	88.0 (84.0–93.0)	89.5 (85.0–94.0)	88.0 (84.0–93.0)	85.0 (82.0–91.0)	0.289	0.147	87.5 (83.8–93.0)	90.0 (84.0–94.0)	0.413
Insulin (IU/L)	5.8 (3.7–8.1)	6.1 (4.4–9.8)	5.8 (3.4–8.1)	4.1 (2.7–5.7)	0.170	0.021	5.6 (3.8–8.1)	6.1 (3.5–10.8)	0.575
HOMA-IR	1.24 (0.80–1.89)	1.43 (0.92–2.12)	1.26 (0.74–1.87)	0.83 (0.58–1.24)	0.109	0.013	1.22 (0.81–1.88)	1.26 (0.76–2.12)	0.588
TG (mg/dL)	82.0 (54.0–118.0)	89.0 (60.3–124.3)	82.0 (54.0–118.3)	49.0 (39.0–87.0)	0.661	0.002	88.5 (58.0–121.8)	67.0 (44.0–94.0)	0.005
T Chol (mg/dL)	171.0 (149.0–187.0)	170.0 (145.8–187.3)	171.5 (150.0–189.3)	163.0 (130.0–181.0)	0.794	0.430	172.0 (151.8–190.0)	162.0 (145.5–177.5)	0.059
HDL-C (mg/dL)	49.0 (42.0–59.0)	51.0 (42.0–59.8)	48.0 (41.8–59.0)	51.0 (45.0–55.0)	0.327	0.822	50.0 (42.8–59.8)	47.0 (39.5–55.0)	0.051
LDL-C (mg/dL)	98.0(84.0–114.0)	97.0 (78.3–111.3)	99.5 (86.0–116.5)	96.0 (76.0–115.0)	0.373	0.840	99.0 (83.8–113.5)	96.0 (86.0–114.0)	0.952
hsCRP (mg/dL)	0.054 (0.010–0.147)	0.046 (0.010–0.097)	0.059 (0.010–0.249)	0.116 (0.036–0.338)	0.144	0.056	0.048 (0.010–0.146)	0.071 (0.025–0.264)	0.129

Data are shown as median (interquartile range) or *n* (%). Abbreviations: BMI, body mass index; WHR, waist-hip ratio; HOMA-IR, homeostatic model assessment for insulin resistance; TG, triglycerides; T Chol, total cholesterol; HDL-C, high-density lipoprotein cholesterol; LDL-C, low-density lipoprotein cholesterol; hsCRP, high-sensitivity C-reactive protein. Data were analyzed using chi-square or Mann-Whitney U test.

^a^ Comparison between VitA normal and risk;

^b^ Comparison between VitA normal and depletion;

^c^ Comparison between VitE normal and deficiency. All statistically significant *p*-values are highlighted in bold.

Notably, VitA depletion was more frequent among underweight and normal weight children as compared to overweight and obesity children. In contrast, the frequency of VitE deficiency was similar across normal weight, overweight, and obesity groups (Supplementary Table 1). Furthermore, the frequency of both VitA depletion and VitE deficiency did not differ significantly by sex (Supplementary Table 2).

### Dietary intake and serum vitamin status

In order to evaluate the influence of diet on vitamin circulating levels, we compared macro and micronutrient consumption among the groups. No significant differences in total kilocalories, carbohydrate, protein, or lipid intake were observed according to VitA and VitE status (Table 2). Although RAE intake was higher in children with normal VitA plasma levels (*p* = 0.061), dietary retinol and carotenes did not differ according to VitA status. Moreover, no significant differences in VitE intake or tocopherol equivalents were observed between children with normal and VitE deficiency levels (Table 2).

**Table 2.** Dietary intake stratified by vitamin A and vitamin E status.

		Vitamin A					Vitamin E		
	All (*n* = 179)	Normal (*n* = 74)	Risk (*n* = 90)	Depletion (*n* = 15)	*p*-value^a^	*p*-value^b^	Normal (*n* = 142)	Deficiency (*n* = 37)	*p*-value^c^
Total energy (Kcal/day)	2041.0 (1516.6–2618.3)	2175.6 (1690.6–2645.2)	1882.8 (1479.0–2558.9)	1933.7 (1426.7–3054.9)	0.145	0.497	2033.3 (1523.0–2629.2)	2099.1 (1471.7–2559.3)	0.956
Proteins (E%)	14.9 (13.6–16.7)	15.4 (13.6–16.9)	14.8 (13.6–16.1)	15.5 (14.2–17.1)	0.301	0.784	14.9 (13.5–16.4)	15.4 (14.2–17.1)	0.090
Lipids (E%)	38.5 (34.6–42.6)	38.3 (35.4–43.6)	38.7 (34.2–42.7)	39.9 (34.9–41.9)	0.583	0.776	38.8 (34.6–43.1)	37.8 (34.7–41.9)	0.755
Carbohydrates (E%)	45.8 (41.5–50.8)	45.3 (40.1–49.4)	46.3 (42.6–51.5)	45.3 (42.4–50.2)	0.181	0.983	45.3 (41.3–50.9)	45.9 (41.9–50.4)	0.342
Micronutrients intake									
Retinol (IU)	2458.3 (1409.3–4577.5)	2804.4 (1605.4–5085.6)	2372.3 (1303.6–4192.0)	2095.3 (1246.5–3852.6)	0.112	0.198	_	_	_
Carotenes (μg)	5843.8 (3513.4–10088.1)	6990.3 (3431.5–10600.9)	4917.6 (3498.3–9582.9)	7294.6 (4293.2–9524.2)	0.350	0.848	_	_	_
RAE (μg)	1083.0 (665.5–1837.6)	1121.8 (813.5–2014.1)	983.1 (571.7–1590.1)	940.5 (598.3–1880.3)	0.061	0.230	_	_	_
Vitamin E (μg)	7.7 (5.8–10.9)	_	_	_	_	_	7.6 (5.7–10.8)	8.7 (6.6–11.6)	0.315
Total tocopherol (mg)	20.5 (15.1–27.7)	_	_	_	_	_	20.5 (15.1–27.3)	20.3 (15.8–30.8)	0.813
Alpha tocopherol (mg)	9.2 (6.6–12.2)	_	_	_	_	_	9.1 (6.4–12.1)	9.9 (6.8–12.5)	0.504

Data are shown as median (interquartile range) or *n* (%). Abbreviations: E, energy; IU, International Units; RAE, Retinol Activity Equivalents. Data were analyzed using Mann-Whitney U test.

^a^ Comparison between VitA normal and risk.

^b^ Comparison between VitA normal and depletion.

^c^ Comparison between VitE normal and deficiency.

### Gut microbiota diversity and composition

Six phyla were identified in the entire study population: Bacteroidota (56.7%) showed the highest abundance, followed by Firmicutes (38.5%), Proteobacteria (3.6%), and Verrucomicrobiota (0.3%) (Supplementary Figure 2A). In addition, 29 different families were identified, where *Bacteroidaceae*, *Lachnospiraceae*, *Prevotellaceae*, and *Ruminococcaceae* were the most abundant (35.7%, 17.3%, 13.8%, and 10.0%, respectively) (Supplementary Figure 2B). Of the 66 genera observed, *Bacteroides* (35.6%), *Prevotella* (12.9%), *Roseburia* (5.6%) and *Faecalibacterium* (4.8%) accounted for more than three quarters of the total bacterial community (Supplementary Figure 2C).

No significant differences in alpha diversity were observed. However, the Shannon index was lower in the risk and depletion as compared to the normal VitA group after being adjusted for age (*p*_*adj*_ = 0.076 and *p*_*adj*_ = 0.052, respectively, Supplementary Figure 3A). Moreover, beta diversity assessed as the weighted UniFrac distance was significantly different among the VitA groups (*p* = 0.03). However, after FDR correction, the pairwise comparisons did not show significant differences (Supplementary Table 3). Regarding VitE, no differences in alpha or beta diversity were observed between the groups (Supplementary Figure 3B and Supplementary Table 3).

Interestingly, the LEfSe analysis showed that four genera were more abundant in children with VitA depletion: *Haemophilus*, which belongs to the Proteobacteria phylum, and *Veillonella*, *Streptococcus*, and *Clostridium sensu stricto 1* from the Firmicutes phylum. In addition, *Lachnoclostridium* was more abundant in children at risk of VitA depletion, while *UCG-005* from the *Oscillospiraceae* family was more abundant in children with normal VitA levels (Figure 1A). However, according to the age-adjusted ANCOM-BC2 analysis, the only four genera more abundant in the VitA depletion group with *q* < 0.2 were *Veillonella* (log FC = 1.43, *q* = 0.07), *Haemophilus* (log FC = 1.41, *q* = 0.14), *Clostridium sensu stricto 1* (log FC = 1.14, *q* = 0.08), and *Streptococcus* (log FC = 1.04, *q* = 0.12) (Figure 1B).

**Figure 1.**
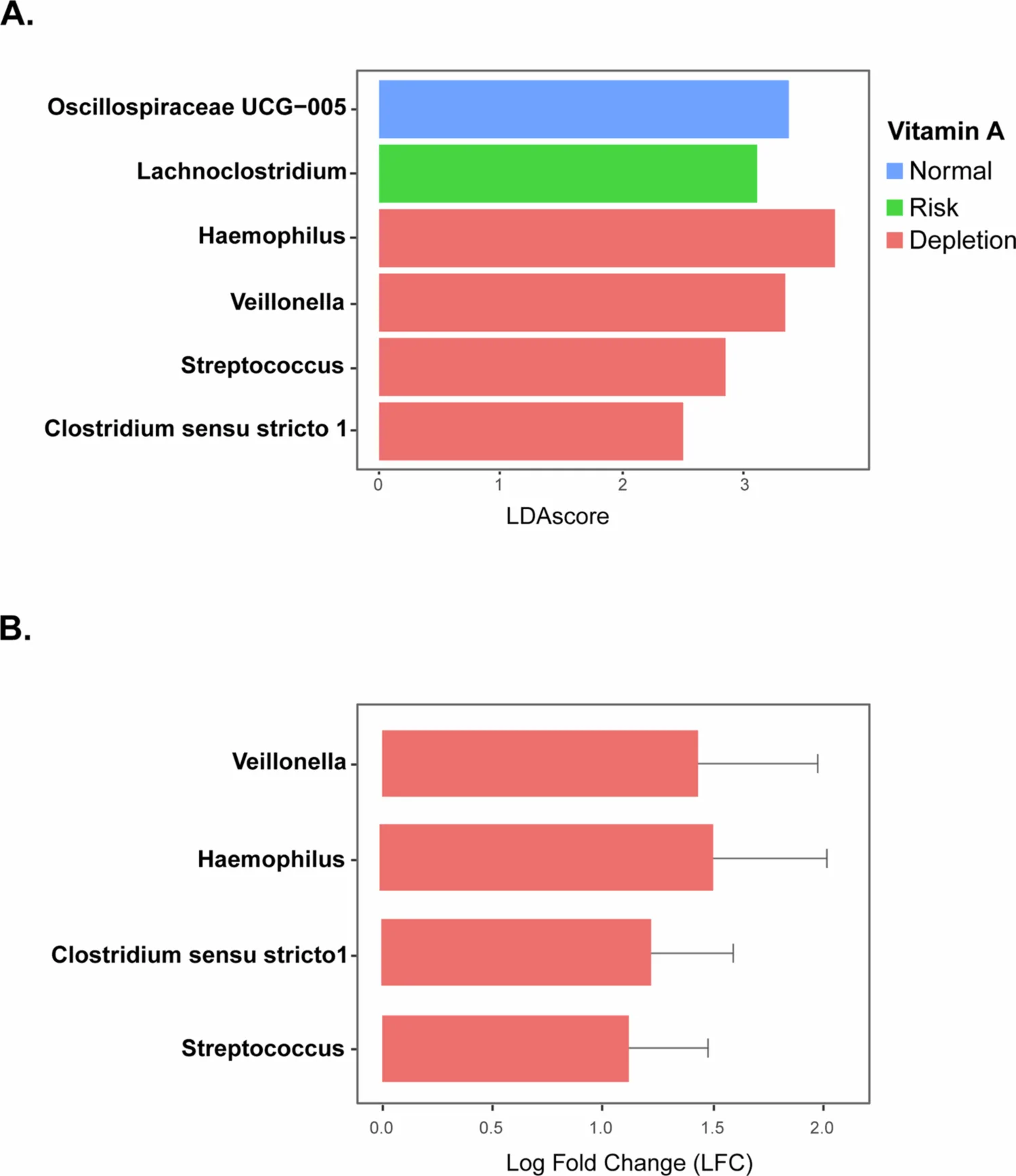
Bacterial genera differentiated according to vitamin A status. (A) LDA score obtained by LEfSE analysis for normal, risk, and vitamin A depletion groups. (B) Log-fold change according to ANCOM-BC2. Only genera significantly differentiated by LEfSE and with a *q*-value < 0.2 are represented.

On stratifying by VitE plasma levels, the Lefse analysis identified five differentially enriched genera from the Firmicutes phylum and *Clostridia* class: *Lachnospira* and *Lachnospiraceae-UCG-004* were more abundant in the VitE deficiency group, while *Oscillibacter, UCG-002*, and *Clostridia-UCG-014* were more abundant in children with normal VitE levels (Figure 2). Nevertheless, the age-adjusted ANCOM-BC2 analysis did not identify *q* values < 0.2 for abundance differences in any of these genera (Supplementary Table 4).

**Figure 2.**
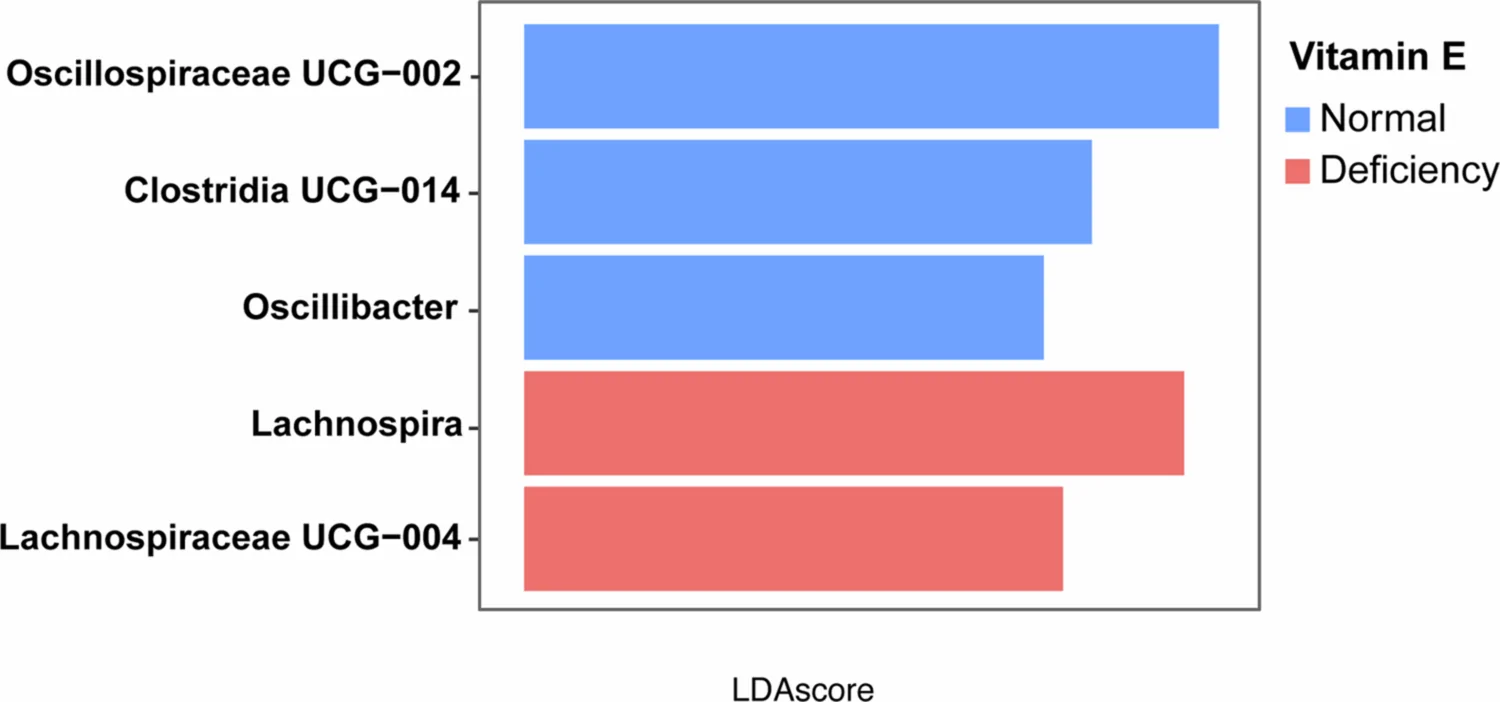
Linear discriminant analysis effect size (LEfSE) at the genus level according to vitamin E status.

### Correlations between genera enriched in VitA depletion and dietary nutrients

We then sought correlations between the abundance of VitA status-differentiated bacteria, with dietary nutrients. Of all macronutrients assessed, only dietary fiber intake correlated negatively with the genus *Veillonella*, enriched in children with VitA depletion levels (Supplementary Figure 4).

Because adequate vitamin intake does not always translate to normal serum vitamin levels, we analyzed the abundance of VitA-depletion-differentiated bacteria only in children with an adequate vitamin A intake (*n* = 154). Interestingly, children with normal VitA intake and VitA depletion showed significantly greater abundance of *Haemophilus*, *Veillonella* and *Streptococcus* (*p* < 0.05) as compared to those with normal VitA levels (Figure 3).

**Figure 3.**
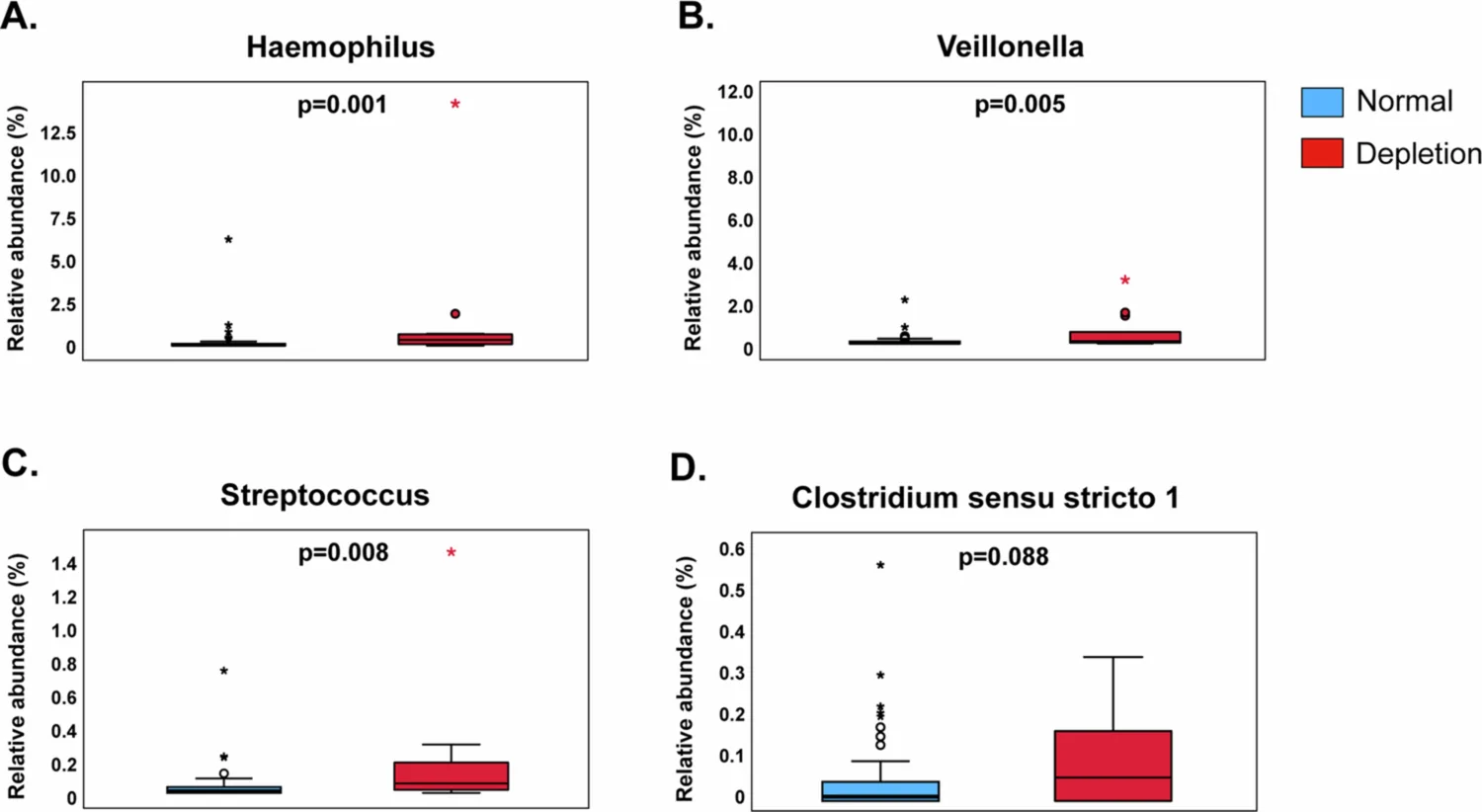
Comparison of the relative abundance of genera identified as enriched in the VitA depletion group, only in children with adequate vitamin A dietary intake.

## Discussion

The present study aimed to identify differences in metabolic traits, dietary intake, and gut microbial composition between children with and without VitA depletion and VitE deficiency. Our approach identified bacterial genera associated with vitamin deficiencies, which may play a role in VitA and VitE plasma level variation and thus impact children's health. Vitamins are essential for many biochemical functions, including energy metabolism, inflammation, and antioxidant activity, and are of utmost importance in growth and health processes during childhood.^40^ Vitamin deficiencies are still common worldwide, particularly in children in developing countries.^41^ ^,^ ^42^ In our group of Mexican children, VitA depletion frequency was below 10%. This is in agreement with studies conducted in other Mexican school-age children cohorts, where the prevalence of low VitA levels ranged between 5% and 7%.^10^ ^,^ ^11^ ^,^ ^43^ ^,^ ^44^ Moreover, 14% of the children from this study had inadequate VitA dietary intake, in accordance with a previous report in Mexican school-age children.^45^ Regarding VitE, inadequate intake and plasma deficiency were common in our study (57.5% and 20.7%, respectively), also coincident with previous studies in Latin and North American children, where VitE deficiency was one of the most common micronutrient deficiencies.^11^ ^,^ ^42^ ^,^ ^45^ ^,^ ^46^

Children with VitA depletion were younger than children with normal VitA levels, as previously reported in several studies.^47^ This could be related to the lower dietary diversity and higher frequency of infections observed at early ages.^48^ ^,^ ^49^ We also found that VitA depletion was more frequent in underweight and normal weight children. These findings are consistent with those of previous studies reporting an association of VitA deficiency with a higher risk of undernutrition, underweight, and stunting in children.^49^ In addition, high VitA dose supplementation in preschool children improved linear growth, particularly when basal serum retinol levels were very low.^50^ Children with VitA risk and depletion also showed higher hsCRP concentrations. A negative correlation between serum retinol levels and hsCRP has been reported in several populations, including other cohorts of Mexican children.^11^ ^,^ ^51^ In contrast, the only difference in biochemical parameters according to VitE status was lower TG levels in children with VitE deficiency. This is in line with VitE being a fat-soluble molecule that depends on dietary lipids and lipoproteins to be absorbed, transported, and stored.^52^

In the present study, the dietary intake (carotenes and tocopherols) was not significantly associated with serum VitA and VitE status. It has been suggested that this lack of correlation could be explained by VitA and VitE hepatic and adipose tissue storage, as these stores can be readily mobilized when serum levels decrease.^53^ ^,^ ^54^ Moreover, recent evidence from clinical studies and animal models suggests that the gut microbiome could be a major determinant of the host vitamin status, influencing processes such as biotransformation, biosynthesis, metabolism, and even the regulation of intestinal absorption.^55^ However, few studies have analyzed the relationship between the gut microbiota and vitamin deficiencies in children,^20^ ^,^ ^56^ ^,^ ^57^ none of which have included Latino populations.

In our study, differences in gut microbiota alpha diversity according to vitamin A and E status did not reach statistical significance. Nevertheless, the Shannon index was lower in children with risk (*p* = 0.07) and VitA depletion (*p* = 0.05) as compared to those with normal VitA levels, in line with previous studies in both mice and humans with vitamin deficiencies.^58-60^

We identified a high abundance of *Lachnospiraceae UCG-004* and *Lachnospira* in children with VitE deficiency by LEfSe analysis, but not by ANCOM-BC2. To our knowledge, *Lachnospiraceae UCG-004* has not been previously related to VitE deficiency. Evidence from murine models suggests that the *Lachnospiraceae* family uses tocopherols as a substrate.^61^ Moreover, in humans, VitE supplementation appears to promote *Lachnospiraceae* proliferation, which could decrease VitE absorption.^62^ ^,^ ^63^ Further studies are needed to assess whether the presence of these bacteria in the gut affects VitE absorption and circulating levels in humans.

Notably, we did not identify bacteria known to be involved in VitA biotransformation in any of our study groups.^16^ ^,^ ^64^ However, LEfSe and ANCOM-BC2 showed significantly higher abundance of *Haemophilus, Veillonella, Streptococcus,* and *Clostridium sensu stricto 1* in children with VitA depletion. This differs from findings in Cambodian children, where *Gordonibacter, Lactobacillaceae* and *Romboustia* were more abundant in children with VitA deficiency.^60^ These differences may be due to environmental and geographical factors, as well as the severity of VitA status (depletion vs deficiency).

Interestingly, in a study of stunted children from Africa, the gut microbiota was characterized by overrepresentation of oral bacteria, including *Haemophilus, Veillonella*, and *Streptococcus*, and decreased abundance of butyrate-producing taxa, considered an intestinal hallmark of stunting.^65^ Although our study did not include stunted children (height-for-age *z*-score ≤ −3 SD), we also observed overrepresentation of these three genera in children with VitA depletion, who showed lower height percentile as compared to normal VitA children. The overgrowth of oral bacteria in the gut of stunted children has been attributed to a decompartmentalization of the gastrointestinal tract that involves the carrying of oropharyngeal bacteria and pathobionts through the stomach, small intestine, and colon, which may be responsible for local damage and inflammation.^65^ Whether this decompartmentalization occurs in children with VitA deficiency remains to be determined.

A higher relative abundance of *Haemophilus* and *Veillonella* has also been reported in young adults with vitamin D deficiency. The authors suggested that these genera could have a competitive advantage in environments with impaired immune response,^66^ a condition also described in VitA deficiency.^67^ Although the mechanism is not fully understood, several lines of evidence suggest that VitA deficiency is related to altered immune responses. Animal models have shown that VitA deficiency or low retinoic acid synthesis enzyme activity is consistently correlated with reduced luminal immunoglobulin A (IgA) and increased bacterial translocation.^68-71^ Moreover, retinoic acid exerts immunomodulatory functions that include guiding IgA-producing B cells to the intestinal mucosa.^72-74^ Thus, it is tempting to speculate that the higher abundance of oral bacteria in children with VitA depletion may also be mediated by decreased secretory IgA levels within the intestinal lumen impairing the immune exclusion process, which normally limits the adhesion of opportunistic pathogens.

Certain limitations of this study should be acknowledged. Firstly, the frequency of VitA depletion and VitE deficiency in our study was small. Moreover, because this study was nested within an existing pediatric cohort, the final analytical sample was restricted only to children with complete data. Consequently, we acknowledge the lack of an a priori microbiome-specific power calculation as a limitation. Additionally, FFQ used as an instrument to assess habitual diet, including micronutrients, may contribute to the overestimation of nutrient intake. Owing to the cross-sectional design of our study, we cannot establish a causal relationship between the gut microbiota profiles and vitamin deficiencies. Whether vitamin deficiencies drive dysbiosis or if a gut microbial imbalance contributes to these deficiencies remains unclear. Therefore, longitudinal or interventional studies are required to elucidate the precise causal mechanisms underlying this bidirectional relationship. Moreover, metagenomic studies are required for a more accurate analysis of bacterial functionality. Finally, because the fecal concentrations of vitamins were not quantified, we have no objective measurement of the gut availability of these vitamins.

In conclusion, to our knowledge, this is the first study to analyze the relationship between the gut microbiota and VitA depletion and VitE deficiency in Mexican school-age children. While no significant associations were observed between VitE deficiency and the gut microbiome profile, VitA depletion was distinctly characterized by an enrichment of oropharyngeal genera, including *Haemophilus, Veillonella*, and *Streptococcus.* This microbial signature closely resembles the gut microbiome composition previously described in malnutrition. Consequently, future studies are required to elucidate whether these specific bacteria play a functional role in vitamin A metabolism.

## Supplementary Material

Supplementary_Figure_1_24sept26.tif Supplementary_Figure_1_24sept26.tif

Supplementary_Table_4.docx Supplementary_Table_4.docx

Supplementary_Table_2.docx Supplementary_Table_2.docx

Supplementary_Table_3.docx Supplementary_Table_3.docx

Supplementary_Figure_3_24sep26.tif Supplementary_Figure_3_24sep26.tif

Supplementary_Figure_4_24sep26.tif Supplementary_Figure_4_24sep26.tif

Supplementary_Figure_2_24sep26.tif Supplementary_Figure_2_24sep26.tif

Supplementary_Table_1.docx Supplementary_Table_1.docx

Supplementary_Figure_captions_.docx Supplementary_Figure_captions_.docx

## Data Availability

All sequencing data that support the findings of this study are available in the NCBI BioProject database under accession number: PRJNA1276625 at http://www.ncbi.nlm.nih.gov/bioproject/1276625.
