# Supplementary material for: Gut microbiota profile in school-aged children with vitamin A depletion and vitamin E deficiency: A cross-sectional study
Source: Gut Microbes Rep. 2026 Oct 16;4(1):2747503. doi: 10.1080/29933935.2026.2747503 (PMC13682405; doi:10.1080/29933935.2026.2747503)
Supplement: Supplementary file 1 [file KGMR_A_2747503_SM3241.docx]

**Supplementary Figure 1.** Rarefaction curves showing observed richness (number of ASVs) as a function of sequencing depth in the 179 samples. Each color line represents a sample. Curves were generated at the amplicon sequence variant (ASV) level. The plateau in most curves indicates sufficient sequencing coverage for capturing microbial diversity.

**Supplementary Figure 2.** Relative abundance at phylum (A), family (B) and genus (C) level in the total study population.

**Supplementary Figure 3.** Comparisons of alpha diversity indices between VitA (A) and VitE (B) study groups. Plotted data represent medians and interquartile ranges; age adjusted *p*-values are shown.

**Supplementary Figure 4**. Heatmap representing the correlation matrix between differentially enriched genera and dietary variables (macro and micronutrients). Spearman correlations were adjusted by sex, age and BMI percentile. *Adjusted *p-*value <0.05. RAE, Retinol Activity Equivalents.
