# Supplementary material for: Gut microbiota profile in school-aged children with vitamin A depletion and vitamin E deficiency: A cross-sectional study
Source: Gut Microbes Rep. 2026 Oct 16;4(1):2747503. doi: 10.1080/29933935.2026.2747503 (PMC13682405; doi:10.1080/29933935.2026.2747503)
Supplement: Supplementary file 2 [file KGMR_A_2747503_SM7174.docx]

| **Supplementary Table 4. ANCOM BC2 results for the taxa differentiated by LEfSe between Vitamin E groups** | | | | | |  |
| --- | --- | --- | --- | --- | --- | --- |
| Taxon | Log Fold Change | SE | W | *p*-value | *q*-value | |
| Oscillospiraceae UCG-002 | -0.434 | 0.289 | -1.503 | 0.135 | 1 | |
| Clostridia UCG-014 | -0.708 | 0.367 | -1.931 | 0.055 | 1 | |
| Oscillibacter | -0.695 | 0.266 | -2.61 | 0.01 | 1 | |
| Lachnospira | 0.476 | 0.313 | 1.52 | 0.13 | 1 | |
| Lachnospiraceae UCG-004 | 0.419 | 0.285 | 1.473 | 0.143 | 1 | |
| Comparisons were adjusted for age. A *q*-value<0.2 was considered significant. | | | | | |  |
