# Supplementary material for: Gut microbiota profile in school-aged children with vitamin A depletion and vitamin E deficiency: A cross-sectional study
Source: Gut Microbes Rep. 2026 Oct 16;4(1):2747503. doi: 10.1080/29933935.2026.2747503 (PMC13682405; doi:10.1080/29933935.2026.2747503)
Supplement: Supplementary file 3 [file KGMR_A_2747503_SM7175.docx]

| **Supplementary Table 2. Vitamin status of the Mexican children cohort stratified by sex** | | | | |
| --- | --- | --- | --- | --- |
| **Trait** | **All**  (n=179) | **Boys**  (n=88) | **Girls**  (n=91) | ***p-value*** |
| **Vitamin concentration** |  |  |  |  |
| Vitamin A (ug/dL) | 28.80 (24.80-33.80) | 28.75 (24.23-34.70) | 28.90 (25.80-32.90) | 0.605 |
| Vitamin E (ug/mL) | 6.80 (5.20-8.10) | 6.70 (5.05-8.20) | 6.80 (5.30-8.10) | 0.814 |
| **Vitamin status** |  |  |  |  |
| Vitamin A risk / depletion, n (%) | 105 (58.7) | 53 (60.2) | 52 (57.1) | 0.675 |
| Vitamin E deficiency, n (%) | 37 (20.7) | 19 (21.6) | 18 (19.8) | 0.765 |
| Data are shown as median (interquartile range) or n (%). Data were analyzed using chi-square or Mann-Whitney U test. *p-*value, comparisons between boys and girls. | | | | |
