# Supplementary material for: Gut microbiota profile in school-aged children with vitamin A depletion and vitamin E deficiency: A cross-sectional study
Source: Gut Microbes Rep. 2026 Oct 16;4(1):2747503. doi: 10.1080/29933935.2026.2747503 (PMC13682405; doi:10.1080/29933935.2026.2747503)
Supplement: Supplementary file 4 [file KGMR_A_2747503_SM7177.docx]

| **Supplementary Table 3. Beta diversity** | | | | | | | | | | | |
| --- | --- | --- | --- | --- | --- | --- | --- | --- | --- | --- | --- |
|  | **Unweighted UniFrac Distance** | | | | **Weighted UniFrac Distance** | | | | | | |
|  | F-value | R squared | *p*-value | *p*-FDR | | | F-value | R squared | | *p*-value | *p*-FDR |
| **Vitamin A** |  |  |  |  | |  | | |  |  |  |
| All groups | 1.081 | 0.012 | 0.213 | - | | 2.280 | | | 0.025 | **0.030** | - |
| Risk vs Normal | 1.226 | 0.008 | 0.080 | 0.240 | | 0.947 | | | 0.006 | 0.416 | 1.000 |
| Risk vs Depletion | 1.004 | 0.010 | 0.425 | 1.000 | | 2.715 | | | 0.026 | **0.038** | 0.114 |
| Normal vs Depletion | 1.134 | 0.013 | 0.163 | 0.489 | | 3.788 | | | 0.042 | **0.027** | 0.081 |
| **Vitamin E** |  |  |  |  | |  | | |  |  |  |
| Normal vs Deficiency | 0.838 | 0.005 | 0.877 | - | | 2.124 | | | 0.012 | 0.069 | - |
| Comparisons were made with PERMANOVA test using adonis2 and adonis.pairwise functions to adjust for age. | | | | | | | | | | | |
