# Supplementary material for: Gut microbiota profile in school-aged children with vitamin A depletion and vitamin E deficiency: A cross-sectional study
Source: Gut Microbes Rep. 2026 Oct 16;4(1):2747503. doi: 10.1080/29933935.2026.2747503 (PMC13682405; doi:10.1080/29933935.2026.2747503)
Supplement: Supplementary file 5 [file KGMR_A_2747503_SM7179.docx]

| **Supplementary Table 1. Vitamin status of the Mexican children cohort stratified by nutritional diagnosis** | | | | | | | | | |
| --- | --- | --- | --- | --- | --- | --- | --- | --- | --- |
| **Trait** | **All**  (n=179) | **Underweight**  (n=8) | **Normal weight**  (n=99) | **Overweight**  (n=36) | **Obesity**  (n=36) | ***p-value^a^*** | ***p-value^b^*** | ***p-value^c^*** | ***p-value^d^*** |
| **Vitamin concentration** |  |  |  |  |  |  |  |  |  |
| Vitamin A (ug/dL) | 28.80 (24.80-33.80) | 32.35 (21.18-35.88) | 28.40 (23.80-31.70) | 31.65 (26.50-41.20) | 27.45 (24.98-34.70) | 0.530 | **0.005** | 0.453 | **0.030** |
| Vitamin E (ug/mL) | 6.80 (5.20-8.10) | 7.05 (6.43-8.05) | 6.80 (5.30-8.20) | 6.85 (5.15-8.68) | 6.20 (4.90-7.95) | 0.366 | 0.765 | 0,192 | 0.380 |
| **Vitamin status** |  |  |  |  |  |  |  |  |  |
| Vit A depletion, n (%) | 15 (8.4) | 1 (12.5) | 12 (12.1) | 0 (0.0) | 2 (5.6) | 0.823 | **0.015** | 0.360 | 0.100 |
| Vitamin E deficiency, n (%) | 37 (20.7) | 0 (0.0) | 20 (20.2) | 7 (19.4) | 10 (27.8) | 0.133 | 0.902 | 0.364 | 0.322 |
| Data are shown as median (interquartile range) or n (%). Data were analyzed using chi-square or Mann-Whitney U test. ^a^Comparison between Normal weight and Underweight; ^b^Comparison between Normal weight and Overweight; ^c^Comparison between Normal weight and Obesity and ^d^Comparison between all groups. All statistically significant *p-*values are highlighted in bold. | | | | | | | | | |
